# Attributing Variance in Supportive Care Needs during Cancer: Culture-Service, and Individual Differences, before Clinical Factors

**DOI:** 10.1371/journal.pone.0065099

**Published:** 2013-05-31

**Authors:** Richard Fielding, Wendy Wing Tak Lam, Shiow Ching Shun, Toru Okuyama, Yeur Hur Lai, Makoto Wada, Tatsuo Akechi, Wylie Wai Yee Li, for Asia-Pacific Psycho-Oncology Network (APPON)

**Affiliations:** 1 Centre for Psycho-Oncology Research and Training, School of Public Health, The University of Hong Kong, Pokfulam, Hong Kong, China; 2 Department of Nursing, National Taiwan University, Taipei, Taiwan; 3 Department of Psychiatry and Cognitive-Behavioral Medicine, Nagoya City University Graduate School of Medical Sciences, Mizuho-cho, Mizuho-ku, Nagoya, Japan; 4 Department of Psycho-Oncology, Saitama Cancer Center, Kita-Adachi-gun, Saitama-ken, Japan; University of Pennsylvania, United States of America

## Abstract

**Background:**

Studies using the Supportive Care Needs Survey (SCNS) report high levels of unmet supportive care needs (SCNs) in psychological and less-so physical & daily living domains, interpreted as reflecting disease/treatment-coping deficits. However, service and culture differences may account for unmet SCNs variability. We explored if service and culture differences better account for observed SCNs patterns.

**Methods:**

Hong Kong (n = 180), Taiwanese (n = 263) and Japanese (n = 109) CRC patients’ top 10 ranked SCNS-34 items were contrasted. Mean SCNS-34 domain scores were compared by sample and treatment status, then adjusted for sample composition, disease stage and treatment status using multivariate hierarchical regression.

**Results:**

All samples were assessed at comparable time-points. SCNs were most prevalent among Japanese and least among Taiwanese patients. Japanese patients emphasized Psychological (domain mean = 40.73) and Health systems and information (HSI) (38.61) SCN domains, whereas Taiwanese and Hong Kong patients emphasized HSI (27.41; 32.92) and Patient care & support (PCS) (19.70; 18.38) SCN domains. Mean Psychological domain scores differed: Hong Kong = 9.72, Taiwan = 17.84 and Japan = 40.73 (p<0.03–0.001, Bonferroni). Other SCN domains differed only between Chinese and Japanese samples (all p<0.001). Treatment status differentiated Taiwanese more starkly than Hong Kong patients. After adjustment, sample origin accounted for most variance in SCN domain scores (p<0.001), followed by age (p = 0.01–0.001) and employment status (p = 0.01–0.001). Treatment status and Disease stage, though retained, accounted for least variance. Overall accounted variance remained low.

**Conclusions:**

Health service and/or cultural influences, age and occupation differences, and less so clinical factors, differentially account for significant variation in published studies of SCNs.

## Introduction

Many cancer patients’ report unmet supportive care needs (SCNs) that seemingly reflect coping deficits in managing cancer diagnosis, disease and treatment. Variation in study results were initially attributed to measurement differences [1] but widespread use of the Supportive Care Needs Survey (SCNS-34) [2], [3] focused research on SCNs variability by tumour type, location and stage, [4], [5] sociodemographic and treatment differences,^5^ and disease trajectory [6], [7].

Single tumour studies, e.g. [8], [9] or larger studies stratified by tumour type, e.g. [4] report predominantly higher unmet Psychological domain SCNs, [4], [5], [8]–[10] usually attributed to disease stage and treatment impacts on daily activities plus concerns over recurrence, and report more sexuality-related SCNs among men than women.e.g. [4] Consistently “psychological factors” (low mood, high anxiety, low satisfaction) and co-morbid symptoms “predict” high levels of reported SCNs, [4], [5], [10] unsurprising if Psychological domain SCNs predominate.

As SCN prevalence studies proliferate, variability attributable to service and cultural factors remain under-investigated, hence several question arise: Are these patterns universal? What are the contributions of clinical services in driving reported SCNs? Are different cultural values important?

Most published SCNs studies report on predominantly white, Anglo-Saxon groups with ‘individualistic” orientations within Australian, North American and North-Western European populations. Asian populations report different USCNs patterns [11]–[13] compared to these “Anglo” samples. [12], [14], [15] This raises doubts about the universality of reported SCN patterns.

One possible explanation is that variation in health care systems can alter diagnostic, treatment and follow-up impacts. However, service variability is largely ignored by the existent SCNs literature. Major cancer centres in wealthier Asian countries generally adhere to the same international guidelines for treatment and related quality control used in major “western” countries. So patients in the published Asian SCNs studies and Australian/European/North American SCNs studies probably received comparable cancer treatments. However these samples differ in terms of the service organization, access and delivery they encountered, which can produce significant care disparities. [16]–[18] Moreover, diverse Chinese patients with colorectal and early-stage breast cancers (ESBC), cared for under different departments of one Hong Kong hospital reported essentially the same SCNs ranking patterns, differing only in ESBC patients reporting more unmet needs. [19] This indicates some hospital-level or more general influences. Hence, predominantly non-disease-related factors must be influencing SCNs. No work we could find has considered SCNs differences by health systems.

Cultural differences in attitudes and responses to cancer and treatment may also explain SCN variation between “east” and “west” [12], [19] as well as “minority” [18], [20] samples. Preference for sources of support and family coping may differ. Different expectations may affect evaluations of clinic support service adequacy, while cultures of service organization and delivery may determine (and be determined by) clinician behaviour.

Combinations of cultural, service and personal factors most likely account for differences in reported SCNs. To test this hypothesis we compared CRC patients from two ethnic groups living in three Asian countries with different health care systems and attempted to partition SCNs differences attributable to service delivery and cultural effects. Adjusting for disease type, severity and treatment enabled us to control for clinical influences; comparing ethnicity can account for some cultural effects while comparison by country can account for differences in health service effects.

If ranking differences occur in different samples of the same cancer type who are receiving comparable treatments, then clinical factors alone are not sufficient to explain the variability.

We hypothesized that if clinical influences were important drivers of USCNs then after adjustment for stage and treatment of disease, the three samples would show comparable USCNs domain scores. If after adjustment for clinical factors, samples differed, then non-clinical factors are implicated. Fewer differences within the same ethnicity than between different ethnicities implicate cultural factors, while differences by place irrespective of ethnicity implicate health service factors.

We estimated cultural factors by anticipating sample comparability in SCN Psychological and Sexuality domain scores. We estimated service factors by anticipating sample comparability on HSI and PCS domain scores, after adjustment for demographic and clinical differences.

## Methods

### Assessment

#### Supportive care needs

The 34-item Supportive Care Needs Survey (short form), assesses patients’ perceived level of unmet supportive care needs across five domains: physical and daily living (PDL) (five items), psychological (PSY) (10 items), patient care and support (PCS) (five items), health systems and information (HSI) (11 items), and sexuality (SEX) (three items). [2], [3], [10] Patients report the magnitude of each specified need over the past month on a 5-point Likert scale (1 = no need, not applicable; 2 = no need, satisfied; 3 = low need; 4 = moderate need; 5 = high need). [2], [3] Unmet need is indicated for item scores of > = 3. A standardized score ranging from 0 (no needs) to 100 (all items high unmet need) is calculated for each domain. The SCNS-34SF Chinese version in Hong Kong, [21] and Taiwanese communities, [22] and the SCNS-34SF Japanese version [23] have good psychometric properties.

Clinical and demographic data were collected from medical records and during interview.

### Subjects and Procedures

Independent studies from Hong Kong (China), Taipei (Taiwan) and Nagoya/Saitama (Japan) contributed CRC patients. All data were collected according to the principles of the Declaration of Helsinki. Local Ethics approval was independently obtained for recruitment and consent procedures from the Nagoya City University Graduate School of Medical Sciences, Saitama Cancer Centre, NTU hospital, and HKU/HA Institutional Review Boards and Ethics committees and all patients gave fully informed written consent for their data to be recorded, stored and used as part of these research studies into the supportive care needs of CRC patients as part of wider examination of clinical needs during cancer. This secondary comparison of pooled data from these studies involved no further data analyses to those already approved by the respective primary study IRBs, and so further independent IRB approval was not deemed necessary.

#### Hong Kong

Consecutive Cantonese- or Mandarin-speaking patients attending Hong Kong University Medical Centre with a confirmed CRC diagnosis aged >17 years, informed of their diagnosis and capable of completing the assessment were enrolled before surgery. Eligible and consenting patients completed follow-up face-to-face interviews with a trained research assistant. SCNS-34 data were collected at 4 months post-surgery during medical oncology out-patient clinic follow-up visits. Clinical and demographic data were obtained from medical records [19].

#### Taiwan

Eligible patients consecutively recruited from the outpatient clinics at oncology and surgical departments of a leading medical centre in northern Taiwan were ≥18 years old, diagnosed with CRC, informed of the diagnosis who were either still receiving active treatment or were post-treatment survivors, able to communicate verbally, who gave written consent after a detailed explanation of the study purposes and procedures. The SCNS-34 data were collected during follow-up out-patient clinic visits for cancer-related treatment, or one month after completion (survivors). Questionnaires were administered by two well-trained research assistants.

#### Japan

Study subjects were ambulatory patients attending outpatient chemotherapy units at Nagoya City University Hospital and Saitama Medical University International Medical Center. Potential participants were randomly sampled from clinic lists using random number tables to control the number of patients enrolled per day. Eligible patients had diagnosis of CRC, age > = 20 years, informed of cancer diagnosis, and capable of completing the survey questionnaire in Japanese. Following informed consent patients completed the self-administered questionnaires at home, returning them the following day. Incomplete answers were clarified by telephone.

### Analysis

Sample datasets were compiled and matched. Coding differences were resolved by discussion with site investigators. Next, samples were compared on demographic and clinical features. Then the top ten unmet needs items were ranked by frequency, and Psychological domain items examined specifically. SCNS-34 standardized domain scores for each sample were calculated. [2], [3] Associations of demographic and clinical variables with standardized domain scores were then examined. Finally, to attribute variance in standardized domain scores, multivariate adjustment was undertaken using hierarchical multiple regression. Three blocks of independent variables associated with, or having *a priori* likelihood of influencing domain scores were entered in the regression analyses. All analyses were performed using SPSS v.19.

## Results

Overall, 552 CRC patients were included in this secondary analysis, 180 from Hong Kong, 263 from Taiwan, and 109 from Japan.

### Sample Comparability

#### Demographic features (Table 1)

The Taiwanese sample was younger than the Hong Kong and Japanese samples, (Bonferroni post-hoc p<0.001), and had the highest educational achievement, followed by Japanese and lastly Hong Kong samples (χ^2^ = 112.8, df 6, p<0.001). More Taiwanese and Japanese patients worked full-time while more Hong Kong participants reported having no job (χ^2^ = 12.44, df 4, p = 0.014). Marital status was comparable across groups.

**Table 1:** Demographic and clinical characteristics of the Hong Kong and Taiwanese samples.

	Hong Kong N %	Taipei N (%)	Nagoya/Saitama N (%)	Difference p
Sample size N	180	263	109	
Age Mean±S.D.	65.88±11.42	58.43±11.02	63.31±9.82	<0.001^1^
Gender				n.s.
Male	111 61.7	150 57	73 67	
Female	69 38.3	113 43	36 33	
Marital status				n.s.
Never married	14 8.1	28 10.6	6 5.5	
Married/cohabiting	132 73.3	210 79.8	88 80.7	
Divorced/Separated	14 7.8	6 2.3	6 5.5	
Widowed	20 11.1	19 7.2	9 8.3	
Formal education				<0.001
None	40 22.2	11 4.2	0 0	
Primary	53 29.4	54 20.5	28 26.2	
Secondary	69 38.3	89 33.8	46 43.0	
Tertiary	18 10.0	109 41.4	33 30.8	
Occupation				0.014
Full-time	42 23.3	78 29.7	32 29.4	
Part-time	8 4.4	14 5.3	10 9.2	
Unemployed	130 72.2*	171 65.0*	67 69.1*	
Time since diagnosis (months) Mean±S.D.	7.9±5			
Stage 0–1				
2				
3/4				
Treatment status (current)				
No active treatment	83 79.8	123 46.8	0 0	
Surgery (this episode)	180 100.0	256 97.3	98 89.9	<0.001
Chemotherapy	81 52.8	73 27.8	109 100	<0.001
Adjuvant	82 45.6	27 10.3	0 0	<0.001
Colostomy	55 69.4	29 11.5	unknown	<0.001
Targetted	2 1.1	22 8.4	0 0	<0.001

* Includes housewives, retired and unemployed. 1. F-test, else Chi Squared.

#### Clinical features (Table 1)

Samples differed by treatment status and proportions of patients with advanced disease. Fewer Japanese patients (∼90%) had received primary surgery compared to 96% and 99% of the Taiwanese and Hong Kong samples respectively (χ^2^ = 19.11 df 2, p<0.001). At the time of completing the SCNS-34 all Japanese patients were receiving chemotherapy compared to 62% of the Taiwanese and 50% of the Hong Kong patients (χ^2^ = 55.73, df2, p<0.001).

### SCN Prevalence and Rankings

Overall fewer Taiwanese than Hong Kong and Japanese patients reported SCNs. The top 10 prevalent SCNs had a frequency of between 17–33% and 18–46% respectively for Taiwanese and HK patients, and 50–70% among Japanese patients (Table 2). However, on average over twice as many Taiwanese, and over three times as many Japanese than Hong Kong patients reported unmet PSY domain SCNs, while the opposite was the case for unmet HSI domain SCNs, which were more prevalent among Hong Kong patients. Japanese patients reported the highest SCNs prevalence across all domains, particularly in PSY and HSI. For Hong Kong patients, HSI and PCS, then PSY domain SCNs were most prevalent. For Taiwanese patients, HSI, Psychological and PDL SCNs were most prevalent. In all three samples the least prevalent domain was SEX, being lowest in the Hong Kong and Taiwanese samples.

**Table 2:** Top-ranked SCNS-34 unmet needs by reported prevalence, by sample: 1. Total sample; 2. Proportions of sample on active treatment, and; 3, who had completed treatment.

	Hong Kong 1		Taiwanese 1		Japanese 2*		Hong Kong 2		Taiwanese 2		Hong Kong 3		Taiwanese 3	
SCNS-34 item	Rank	Unmetneed N(%)	Rank	Unmetneed N(%)	Rank	Unmetneed N(%)	Rank	Unmetneed N(%)	Rank	Unmetneed N(%)	Rank	Unmetneed N(%)	Rank	Unmetneed N(%)
One member of hospital staff withwhom you can talk to …^a^	1	82 (46)	4	60 (23)	16	48 (44)	1	40 (49)	5	34 (34)	2	42 (44)	4	26 (16)
Being informed about cancer whichis under control or diminishing^a^	2	78 (43)	12	39 (15)	13	51 (47)	3	35 (43)	12	23 (23)	1	42 (44)	12	16 (10)
Being informed about things you can do to help yourself get well^a^	3	78 (43)	1	86 (33)	6	65 (60)	2	39 (48)	1	42 (42)	3	38 (40)	1	44 (27)
Being informed about your test results as soon as feasible^a^	4	61 (34)	11	40 (15)	22	44 (40)	6	27 (33)	15	21 (21)	4	34 (36)	10	19 (12)
Being given written information about…your care^a^	5	57 (32)	15	34 (13)	20	44 (40)	4	29 (36)	17	19 (19)	5	28 (29)	13	15 (9)
Being given explanations of those tests for which you would like explanation^a^	6	54 (30)	7	48 (18)	17	47 (43)	5	28 (35)	7	28 (28)	6	26 (27)	8	20 (12)
Being adequately informed about benefits and side effects of treatments (before choosing them)^a^	7	45 (25)	10	44 (17)	21	44 (40)	7	26 (32)	14	21 (21)	7	19 (20)	5	23 (14)
Being treated like a person, not just another case^a^	8	37 (21)	27	23 (9)	24	39 (36)	8	21 (26)	30	9 (9)	12	15 (16)	16	14 (9)
Staff acknowledge feeling & needs^d^	9	36 (20)	28	20 (8)	26	35 (32)	9	18 (22)	26	13 (13)	8	16 (17)	29	7 (4)
Being given information aboutmanaging illness at home^a^	10	33 (18)	3	62 (23)	15	48 (44)	10	18 (22)	11	24 (24)	11	15 (16)	2	38 (23)
Worry results of treatment beyond control^b^	16	35 (9)	2	64 (24)	4	66 (61)	18	6 (7)	3	37 (37)	20	7 (7)	3	27 (16)
Uncertainty about the future^b^	17	32 (9)	5	56 (21)	5	65 (60)	19	6 (7)	2	39 (39)	17	8 (8)	11	17 (10)
Fears about cancer spreading^b^	18	28 (8)	6	55 (21)	1	76 (70)	23	5 (6)	4	34 (34)	19	7 (7)	6	21 (13)
Not able to do the things I used to^c^	19	25 (7)	8	47 (18)	8	58 (53)	17	6 (7)	6	28 (28)	31	2 (2)	9	19 (12)
Concerns about worries of those close to you^b^	24	22 (6)	9	47 (18)	2	72 (66)	31	3 (4)	8	26 (26)	22	7 (7)	7	21 (13)
Anxiety^b^	28	14 (4)	16	31 (12)	3	67 (61)	28	4 (5)	20	17 (17)	25	5 (5)	14	14 (9)
Feelings about death & dying^b^	21	20 (6)	14	37 (14)	7	61 (56)	20	6 (7)	10	25 (25)	21	7 (7)	20	12 (7)
Feeling down or depressed^b^	26	17 (5)	18	30 (11)	9	56 (51)	21	5 (6)	19	18 (18)	23	6 (6)	19	12 (7)
Keeping a positive outlook^b^	31	12 (4)	20	30 (11)	10	55 (50)	24	5 (6)	13	21 (21)	16	9 (9)	26	9 (5)
Lack of energy/tiredness^c^	24	10 (5)	13	37 (14)	14	48 (44)	16	6 (7)	9	25 (25)	29	3 (3)	17	12 (7)
Staff attend promptly to physical needs^d^	11	30 (17)	24	24 (9)	30	28 (26)	12	14 (17)	23	15 (15)	9	16 (17)	27	9 (5)
Access to professional counselling^d^	13	29 (16)	26	23 (9)	18	47 (43)	14	12 (15)	24	15 (15)	10	16 (17)	28	8 (5)

* All patients on active treatment.

a Health information & systems domain;

b Psychological domain.

c Physical & daily living domain.

d Patient care & support domain.

Examining SCNs by frequency indicated that SCN domains emphasized varied by sample origin (Table 2). Among Hong Kong patients the 10 most frequent SCNs were all HSI domain items. Among Taiwanese patients five HSI, four Psychological, and one PDL domain items comprised the top 10 SCNs. In the Japanese sample eight PSY, one HSI and one PDL domain items comprised the top 10 SCNs.

The most prevalent (“top ranked”) Hong Kong SCN achieved 4^th^ rank in the Taiwanese and 16^th^ in the Japanese sample; the most prevalent Taiwanese SCN achieved 3^rd^ rank in the Hong Kong and 6^th^ in the Japanese samples (Table 2). The most prevalent Japanese SCN achieved 18^th^ and 6^th^ rank respectively in the Hong Kong and Taiwanese samples. Japanese and Hong Kong samples shared only one common item in their respective top 10 SCNs, at 6^th^ rank in the Japanese and 3^rd^ in the HK samples, whereas the Taiwanese and Japanese patients shared five common items in their respective top 10 SCNs.

Stratification by treatment status (active chemotherapy/no chemotherapy) (Table 2) changed sample SCNs rankings somewhat. In the Hong Kong sample four items (two PCS, 2 HSI) differed by up to four ranking positions, whereas the Taiwanese sample differed by up to 9 ranking positions. All Japanese patients were receiving chemotherapy hence this analysis was inapplicable.

The proportion of the Hong Kong sample reporting “unmet need” SCNs scores (item scores = >3) differed between 3% (5 items) fewer to 12% more among patients on- versus off-chemotherapy (Table 2). In the Taiwanese sample, between 0–29% more patients on- versus off-chemotherapy reported unmet SCNs corresponding to the top 10 items.

For PSY domain items, proportions of patients on-treatment and off-treatment reporting “No need” (score 1) and “No need, satisfied” (score 2) ranged between 10–68% and 26–84% (Taiwanese) compared to 1–35% and 58–89% respectively for comparable Hong Kong patients. Japanese on-treatment proportion ranges were 15–35% (score 1) and 11–24% (score 2).

### SCNS Domain Scores

Mean SCNS domain scores (Table 3) were similar for Hong Kong and Taiwanese on PCS (18.38 vs. 19.7, n.s.), PDL (11.00 vs. 13.63, n.s.) and SEX domains (2.51 vs. 4.25, n.s.). PSY domain means were ∼70% higher in Taiwanese compared to Hong Kong samples (9.72 vs. 17.84, Bonferroni, p<0.001) whilst HSI domain scores were ∼20% higher in Hong Kong compared to Taiwanese samples (32.92 vs. 27.41; Bonferroni, p = 0.027). Hong Kong and Japanese samples indicated similar HSI domain scores (32.92 vs. 38.61, n.s.) but Japanese scores were significantly higher than Hong Kong scores for the remaining domains (Bonferroni p all <0.001). Japanese scores significantly exceeded Taiwanese scores for all domains (Bonferroni p all <0.001) (Table 3).

**Table 3:** Mean standardized SCNS-34 domain scores, 1. Hong Kong, 2. Taiwan and 3. Japan.

SCNS-34 domain	Hong Kong^1^ Meanscore	S.D.	Taiwanese^2^ Meanscore	S.D	Japanese^3^ Meanscore	S.D.
Health System & Information^a^	32.92	24.20	27.41	18.02	38.61	25.08
Psychological^b^	9.72	14.50	17.84	17.15	40.73	27.27
Physical & Daily Living^c^	11.00	13.01	13.63	14.74	26.83	21.39
Patient Care & Support^d^	18.38	18.82	19.70	15.40	31.38	21.50
Sexuality^e^	2.51	8.09	4.25	11.74	14.37	12.92

a F = 10.94, df 2,550 p<0.001. Bonferroni tests 1–2 p = 0.027, 2–3 p<0.001, 1–3 n.s.;

b F = 94.42 df 2,551 p<0.001. Bonferroni 1–2 p<0.001, 2–3 p<0.001, 1–3 p<0.001;

c F = 37.21 df 2,550 p<0.001. Bonferroni 1–2 n.s., 2–3 p<0.001, 1–3 p<0.001;

d F = 20.69 df 2,550 p<0.001. Bonferroni 1–2 n.s., 2–3 p<0.001, 1–3 p<0.001;

e F = 29.73 df 2,550 p<0.001. Bonferroni 1–2 n.s., 2–3 p<0.001, 1–3 p<0.001.

Next, mean domain scores fully adjusted by gender, age, education, employment status (Block 1 Demographics), treatment status (chemotherapy/no chemotherapy), disease stage (early/late), (Block 2 Clinical), and sample (Taiwan (referent), Hong Kong, Japan) (Block 3 Origin) were compared using Hierarchical regression. Variables were stepwise entered in blocks 1–3 to determine the relative contribution of each set of variables to domain score differences. Each SCNS domain was tested independently.

#### Health system & information

Origin (Block 3, F = 19.868, df 2,534, p<0.001) and Demographics, (Block 1, F = 2.15, df 6, 538, p = 0.046) significantly increased the explained variance in HSI domain scores. The final model (Table 4) retained sample origin (Hong Kong β = −.180, t = −3.716, p<0.001; Japan β =  = −.279, t = −5.956, p<0.001 referent Taiwan), age (β = −.192, t = −3.863, p<0.001), employment (β = .126, t = 2.677, p = 0.008), and treatment status (β = .103, t = 2.304, p = .022). Younger patients, those in full-time employment, on active treatment and from Hong Kong or Japan had higher standardized HSI SCNs domain scores. However, the final model accounted for only 8.6% of variance in HSI scores.

**Table 4:** Final Hierarchical model of standardized domain scores regressed on demographics (block 1), clinical (block 2) and sample origin (block 3).

USCN Domain	Health System & Information				Psychological				Patient Care & Support				Physical & Daily Living				Sexuality			
Model 3	B	S.E.	β	t	B	S.E.	β	t	B	S.E.	β	t	B	S.E.	β	t	B	S.E.	β	t
Age	−.371	.096	−.192	−3.86†	−.342	.083	−.180	−4.13†	−.271	.080	−.167	−3.39**	−.167	.070	−.114	−2.39**	−.166	.060	−.135	−2.78**
Education^1^ (None/Primary)	1.914	3.626	.025	.53	−4.966	3.124	−.065	−1.59	−.589	3.012	−.009	−.20	−2.556	2.636	−.044	−.97	1.453	2.245	.030	.65
(Secondary)	.394	2.419	.008	.16	−1.824	2.084	−.036	−.87	2.651	2.009	.061	1.32	.430	1.761	.011	.24	1.087	1.498	.033	.73
(Tertiary)	−2.529	2.358	−.052	−1.07	−1.075	2.032	−.022	−.53	−1.812	1.959	−.045	−.93	−.240	1.1714	−.006	−.14	−.509	1.460	−.016	−.35
Occupation^2^ (Part−time)	.038	3.974	.000	.01	3.119	3.424	.034	.911	3.738	3.301	.047	1.13	2.571	2.888	.036	.89	−5.312	2.461	−.089	−2.16*
(Full−time)	6.249	2.334	.126	2.68**	5.350	2.011	.110	2.66**	2.804	1.939	.068	1.45	5.00	1.697	.133	2.95**	−1.572	1.445	−.050	−1.09
Treatment (Active)	4.845	2.103	.103	2.30*	5.696	1.812	.123	3.14†	2.278	1.747	.058	1.30	4.998	1.530	.140	3.27**	.796	1.302	.027	.61
Stage (Advanced)	.025	.021	.048	1.16	.031	.019	.060	1.67	.021	.081	.049	1.20	.035	.016	.089	2.25*	−.004	.013	−.013	−.31
Sample^3^ (Hong Kong)	−8.533	2.296	−.180	−3.72†	7.477	1.978	.160	3.78†	−.863	1.907	−.022	−.45	2.80	1.671	.078	1.68	−.087	1.422	−.003	−.06
(Japan)	−15.591	2.618	−.279	−5.96†	−27.67	2.255	−.501	−12.27†	−14.835	2.174	−.316	−6.82†	−16.57	1.903	−.390	−8.71†	−11.498	1.621	−.324	−7.09†

1 Referent: Secondary;

2 Referent: Not employed;

3 Referent: Taiwanese sample.

* p = 0.03–0.02,

** p = 0.01–0.001,

† p<0.001.

#### Psychological

Origin (F = 104.952, df 2,534, p<0.001) and Demographics (F = 3.128, df 6,538, p = 0.005) significantly increased the explained variance in Psychological domain scores. The final model retained sample origin (Japan β = −.501, t = −12.268, p<0.001; Hong Kong β = .160, t = −3.779), age (β = −.180, t = −4.134, p<0.001), treatment status (β = .123, t = 3.144, p = .002) and employment (β = .110, t = 2.66, p = 0.008). Taiwanese and Japanese patients, younger and on active treatment and patients in full−time employment reported higher PSY SCNs domain scores. The final model accounted only for 9% of score variance.

#### Patient care & support

Origin (F = 24.577, df2,536, p<0.001) and Demographics (F = 2.825, df6,538, p = 0.01) significantly increased the explained accounted variance in PCS domain scores. Only sample origin (Japan β = −.316, t = −6.822, p<0.001) and age (β = −.167, t = −3.389, p = 0.001) were significant in the final model, accounting for 13% of score variance. Japanese and younger patients had higher PCS domain scores.

#### Physical & daily living

Only Origin (F = 47.711, df 2,533, p<0.001) significantly increased the explained variance in standardized PDL domain scores. The final model retained sample origin (β = −.390, t = −8.711, p<0.001), treatment status (β = .140, t = 3.266, p = 0.001), employment (β = .133, t = 2.947, p = 0.003), age (β = −.114, t = −2.395, p = 0.001) and disease stage (β = .089, t = 2.249, p = 0.025), accounting for 3.1% of variance. Japanese patients, those on treatment, working full-time, younger, and with metastatic disease recorded significantly higher domain scores.

#### Sexuality

Origin (F = 27.380, df 2,534, p<0.001) and Demographics (F = 4.656, df 6,538, p<0.001) significantly increased explained variance in Sexuality domain scores. The combined model retained sample origin (β =  −.324, t = −7.094, p<0.001), age (β =  −.135, t =  −2.785, p = 0.006), and employment (β =  −.089, t =  −2.159, p = 0.031) accounting for only 1.6% of domain score variance. Japanese patients, those younger, and employed part-time reported higher SEX domain needs scores, but accounted for just 1.6% of explained variance.

## Discussion

This study compared one Japanese and two different Chinese CRC patient samples. All data were collected by comparable methods, between ∼4–6 months following diagnosis. Nonetheless, samples differed by demographics, disease stage and treatment status. The challenge was to control these differences and explain any residual observed SCNs variation.

All participants came from one of two different cultural and three health service settings. The observed SCNs reflected distinctly different sets of concerns among each sample. For Japanese patients, PSY, followed by HSI domain SCNs were paramount; for Hong Kong patients HSI SCNs overshadowed all other domains. Taiwanese patients also emphasized HSI SCNs, but had the lowest HSI mean score. Otherwise they were consistently placed in between the other two samples, obtaining SCNs scores more similar to the Hong Kong than Japanese group. Marked differences in HSI scores suggest differences primarily in information preference, reflecting variability in either information provision and/or expectation thereof.

Each sample produced markedly different SCNs prevalence-rankings, as anticipated indicating that SCNs patterns are not merely a function of different cancer types. SCNs ranking and score varied by treatment-status, but as hypothesized, also interacted with sample origin. Treatment status markedly differentiated Taiwanese but barely differentiated Hong Kong patients. Taiwanese patients off-chemotherapy showed lower PSY and HSI domain needs. Taiwanese patients on chemotherapy nonetheless reported psychological SCNs levels of only around one third to one half of those reported by Japanese patients; Hong Kong patients on chemotherapy reported levels around ∼10% of those reported by Japanese patients. Comparing “no need” and “need satisfied” scores revealed many more Hong Kong than Taiwanese or Japanese patients reported “No need” on PSY domain items. These data suggest service or more likely, cultural differences, with more “need satisfied” scores in Hong Kong and Taiwan than Japan probably reflecting variation in effectiveness of family and clinical support.

To accommodate culture, we hypothesized that the two Chinese samples would be comparable but differ from the Japanese sample most visibly in PSY and SEX domain scores. This was so for SEX domain scores, probably reflecting different cultural attitudes regarding sexuality between Confucian Chinese and non-Confucian Japanese samples. Yet all three groups reported markedly lower SEX scores than previously reported for European samples (Table 5). PSY domain scores differed between all three groups, by up to a factor of four, again indicating most probably service interacting with cultural and demographic differences. Surprisingly, among these Japanese CRC patients, of 10 top ranked SCNs seven correspond to the top 10 items ranked by a separate sample of Japanese ESBC patients. [13] This parallels the high degree of correspondence seen in the top 10 SCNs rankings by independent samples of Hong Kong breast and CRC patients. [19] In contrast, the Taiwanese and Hong Kong CRC samples respectively ranked 6 and 2 of the same top 10 SCNs as Japanese early stage breast cancer (ESBC) patients. [13] Among 1,250 Korean women with breast cancer HSI domain SCNs predominated. [11] Their top 10 SCNs corresponded to 10 of the top 11 SCNs reported by as sample of Chinese women with ESBC, [12] whereas the top 10 SCNs among similar German ESBC^12^ patients’ matched only 5 of the Korean top 10, [11] and, despite being quite closely matched the German sample shared only 4 top 10 ranked SCN items with a Chinese ESBC sample. [12] Among Japanese [13] ambulatory ESBC patients only four top 10 SCNs were HSI items corresponding to the top 10 items of a Chinese ESBC sample [12] while five (3 HSI, 2 PSY) corresponded to top 10 ranked German items. [12] The remaining four top 10 Japanese items were all PSY domain needs.

**Table 5:** Mean standardized SNCS domain scores for three western and two Asian samples.

	Lam et al Chinese BC [12]	Akechi et al, Japanese BC [13]	Knobf et al USA* Mixed [14]	Lam et al German BC [12]	Bradart et al, French Mixed [15]
Health System & Information (HSI)	47.60	37.1	23.88	32.75	32.54
Psychological	16.51	33.32	31.33	30.70	37.7
Physical & Daily Living (PDL)	16.34	20.90	27.61	26.31	34.42
Patient Care & Support (PCS)	29.20	28.07	21.00	20.35	25.28
Sexuality	5.46	11.50	24.57	19.96	28.92

* SCNF-59, else SCNS-34.

BC = Breast cancer. Mixed = patients with varied cancer sites.

Table 5 compares mean standardized SCNS-SCNs domain scores reported by German, [12] Chinese, [12] and Japanese [13] ESBC patients, [12] to two large samples of mixed cancer patients from Connecticut, USA, [14] and France/Switzerland. [15] Together with the present study, these data incontrovertibly indicate that some aspect of place – culture, health services or both - strongly influences SCNS scores, independently of disease characteristics.

Finally, we hypothesized that comparable HSI and PCS domain scores after adjustment would exclude service as influencing SCN scores. While HSI domain scores were similar and significantly higher among Japanese and Hong Kong than Taiwanese samples, Japanese PCS scores differed from both Chinese samples’ PCS scores, which were comparable. This implicates health service/“care culture” influences.

Consistent with studies elsewhere, [2], [4], [7], [14], [15] age and full-time occupation also influenced HSI, PSY and PDL domain scores. Working and coping with cancer probably increases psychological demands and hence need, particular for information, while worry is likely regarding disease interference with work, financial and family security in younger patients with responsibilities.

Only in HSI and PSY domain scores did Hong Kong and Taiwanese samples differ notably. The Japanese sample differed from the Taiwanese in all five domains and from the Hong Kong sample on four domains. HSI and PSY domain differences implicate cultural and/or service influences affecting CRC’s meaning and/or impact, and service providers’ roles, possibly interacting with age, working status and probably clinical factors, affecting SCNs reporting. Japanese patients had greater psychological needs than all Chinese patients, while Hong Kong patients reported remarkably few psychological needs.

What explains these differences? Lack of support services is unlikely. Japan has probably the most well-developed psychooncology support services in Asia. [24] Hong Kong tertiary hospitals in contrast have modestly-resourced, NGO-funded Cancer Patient Resource Centres, but referral rates by clinicians to these centres’ social workers remain low. Doctors themselves provide little support in high-throughput clinics.

Traditionally in Japan most CRC patients are seen by surgeons who also oversee chemotherapy and other treatment. Nowadays attending physicians in some hospitals transfer patients to medical oncologists for chemotherapy. Nagoya City University hospital adopts the former, and Saitama the latter system. In Taiwan, most cancer patients visit oncologists at medical centres where the average 5–10 minute consultation time limits explanation and support. Although cancer care managers offer education materials and consultation service in clinical settings, the ratio of patient to case managers is too high to be of much practical benefit. Care in Hong Kong involves surgeons disclosing diagnosis and initiating surgery but medical oncologists manage patients subsequently. Public hospital clinics use “supermarket-checkout” queuing with the next patient in line seeing the next available doctor. Clinic loads and hence consultation times are comparable to those in Taiwan. Hong Kong Chinese people are highly pragmatic prioritizing return to normalcy and harmony during cancer. [25] They view surgeons’ and oncologists’ roles as limited to diagnostics and therapeutics, and clinicians mostly concur.

Why might Japanese patients report much higher SCNs? Among young Japanese balanced reciprocity of support is important. [26] Receiving more support from others than was provided in return (overbenefitting) generated feelings of indebtedness, associated with better mental health. However, if less support than requested is received, poorer mental health was seen. [26] Caucasians privilege cultural values that emphasize independence over maintaining social order. [27], [28] Independence, in a western sense, is difficult in Japan as “independent interdependence” (*Jiritsu*) dictates Japanese interactions, and reciprocity helps maintain harmony (*wa*). [29] Social hierarchy warrants communication using polite deference (*enryo*), avoiding offence to higher status persons. Help is sought within ones intimate social group where emotion expression and help-seeking (*honne*) is expected and support (*amae*) provided. [30] *Enryo* may inhibit *honne* during clinical interactions. Familial support should compensate, but perhaps to avoid indebtedness Japanese cancer patients minimize help-seeking from others. Non-symmetrical support exchanges are associated with loneliness and dissatisfaction. [31], [32] This may increase psychological isolation at a time of significant stress. Alternatively, to preserve *wa* in domestic relationships and avoid overburdening family, cancer patients may anticipate greater psychological support from clinicians who, assuming domestic support avoid *amae,* thereby inadvertently psychologically isolating patients [33].

Maintaining harmony dominates Chinese culture, particularly within families. [34], [35] Disease threatens family harmony thereby generating significant coping demand. Traditional family responses involve “protecting” the patient by withholding information and pretending “normality”, but current attitudes mostly favour fully-informing the patient and the family. [34] Fatalism may account for higher psychological needs among Taiwanese [36] while pragmatism may protect Hong Kong patients, possibly explaining very low reported SCNs.

SCNs, principally psychological needs [37] remain high especially among younger patients. Younger age was also an important predictor of unmet need in two Australian CRC patient cohorts [38], [39], but in contrast, ∼50% or more of those cohorts’ patients reported predominantly physical domain SCNs.

Low SEX scores are unlikely to reflect artefact from unwillingness to discuss sexuality. Hong Kong Chinese women with ESBC show similar low unmet needs scores. [19] Such women do not have problems discussing sexuality, rather they tend not to emphasize sexuality as a pressing need or, report having sexuality needs met. [40] Japan has a more liberal sexuality ethic than do Confucian Chinese cultures, and this may in part account for the slightly higher scores seen in the Japanese CRC sample, which however remain below those of “Anglo” samples.e.g. [14], [15] Most of the patients in these samples were older adult males, and age may have contributed to lower sexuality needs.

To summarize, three lines of evidence support our case: First, after adjustment for differences comparable samples of Hong Kong and German women with early stage breast cancer report divergent patterns of SCNs; German women report greater PSY and SEX SCNs, and Hong Kong women greater HSI SCNs. [12] Second, two dissimilar Hong Kong Chinese samples, women with ESBC and older, mostly male CRC patients reported convergent SCN patterns [20]; likewise, dissimilar Japanese CRC and ESBC patients also show very similar SCNs patterns. [13] Third, we have shown that Chinese CRC groups differed in HSI and PSY needs, but not PCS, PDL or SEX needs: in contrast both Chinese CRC samples differed in four of five SCNs domains from Japanese CRC patients. Chemotherapy and disease stage remained minor influences. Taken together these data implicate interacting cultural/service and demographic differences and then clinical factors best account for SCNs differences between published studies.

Study limitations include incomplete adjustment for all potential influences on SCNs, including time since surgery and concurrent symptoms, unavailable for some samples. Operationalization of service and culture influences was imperfect. Time of data collection differed between the samples. Trajectory therefore remains unaccounted for. Measures of support and cultural attitudes towards cancer were unavailable. Hence, fully disambiguating service from cultural factors was not possible with our data. Finally, accounted variance was low. However, we believe this is the first published decomposition of SCNs variance and results are consistent with cultural and service differences outweighing clinical factors in reported variance seen in SCNS-34 patterns.
